# Sensory Brand Experience: Development and Validation in the Chinese Context

**DOI:** 10.3389/fpsyg.2020.01436

**Published:** 2020-07-21

**Authors:** Fang Gao, Xiaomin Lan

**Affiliations:** ^1^Research Center of City Development, Shanghai Urban Construction Vocational College, Shanghai, China; ^2^Institute for Nation(al) Branding Strategy, East China Normal University, Shanghai, China

**Keywords:** sensory brand experience, brand experience, sensory branding, scale development, scale validation

## Abstract

Extant scales related to measuring the sensory aspect of a brand from the consumer’s perspective are typically either too abstract or too concrete. Thus, this study aimed to create a scale with a medium degree of abstraction by which to measure sensory brand experience. This entailed a process of scale development and validation. In study 1, we conducted a qualitative study to explore possible dimensions and items using semi-structured interviews. Several dimensions and items were proposed by combining findings from a literature review and the consumer interviews. In study 2, we examined the items and preliminarily tested the validity of the scale. The results show that, according to our scale, most of the brands considered could be differentiated in terms of the sensory experience they generate. The scale is thus deemed to have potential as a useful tool by which to evaluate the sensory quality of brands. In study 3, we further examined the items, verified the dimensions, tested the reliability and validity of the scale, and formally presented a final version of the scale. This final version comprises three dimensions and 10 items. The three dimensions represent, respectively, three important factors that may influence consumers’ perceptions and evaluations of the sensory quality of brands: the volume of sensory brand stimuli, the uniqueness of sensory brand stimuli, and the consistency between sensory brand stimuli and consumer. The scale’s reliability and validity are found to be satisfactory. Future research can thus employ the scale to assess the sensory experience of various brands, and even to rank brands accordingly. While the present study in the Chinese context is expected to provide valuable insights into the brand experience and sensory branding literature, further research could be conducted to validate the scale in other geographical and cultural contexts.

## Introduction

Over the last decade, brand experience has been a significant and promising construct in consumer research. In addition, brand experience is playing an increasingly important role in managerial practices to build brands. This concept is gaining attention from marketing scholars and practitioners (Tsai, 2005; Brakus et al., 2009). However, brand experiences can differ significantly in terms of their strength and intensity (Brakus et al., 2009; Zarantonello and Schmitt, 2013). For example, Starbucks and Apple have often been mentioned as brands that provide strong and memorable experiences, while Macy’s and Dell have been highlighted as brands that provide only weak experiences (e.g., Brakus et al., 2009; Hultén, 2011). Experiential brands tend to yield several marketing advantages, including enhanced customer satisfaction, loyalty, brand equity, brand personality, and strong brand–consumer relationships. Thus, in an increasingly competitive business environment, organizations must focus on improving brand experiences (Frow and Payne, 2007).

Brand experience is defined as consumers’ subjective internal responses (sensations, feelings, and cognitions) and behavioral responses induced at different levels of interaction, both direct and indirect, with brand-related stimuli (Meyer and Schwager, 2007; Brakus et al., 2009). When consumers search for, buy, and consume brands, they are exposed to various stimuli related to those brands. Such stimuli may be part of a brand’s design and identity (e.g., name, logo, signage), packaging, marketing communications (e.g., advertisements, brochures, Web sites), and environments in which the brand is marketed or sold (e.g., stores, events) (Brakus et al., 2009). Due to brand stimuli, consumers form certain brand perceptions after experiencing a brand. Among the various brand stimuli, sensory stimuli are primary. For brand management, appealing to the five human senses (sight, hearing, touch, smell, and taste) has great potential to establish strong and unique impressions in consumers’ minds (Hultén, 2011). Sensory brand experience is evoked directly by sensory brand-related stimuli, primarily comprising visual, auditory, tactile, olfactory, and gustatory stimuli, and captures aesthetic and sensory qualities that appeal to the senses (Zarantonello and Schmitt, 2010; Nysveen et al., 2013). Recently, sensory brand experience has received increasing attention from researchers, who have stated that sensory brand experience can promote a brand’s value-generation process and equity (Hultén et al., 2009; Hultén, 2011; Lin, 2015; Moreira et al., 2017).

While the aforementioned definition and operationalization of the brand experience construct provides a solid foundation on which to measure the sensory aspect of brands, they are inadequate for guiding the management practice of sensory branding. This is primarily because Brakus et al.’s (2009) measure only captures the presence of brand experiences and does not provide more specific information about the brand’s benefits and attributes (Cho et al., 2015). In other words, it is too abstract for sensory branding practice, since it is an outcome-oriented, rather than process-oriented measure. In fact, Brakus et al.’s (2009) measure of sensory dimension comprises only three items: “This brand makes a strong impression on my visual sense or other senses,” “I find this brand interesting in a sensory way,” and “This brand does not appeal to my senses.” Nevertheless, a thorough literature review of papers on “brand experience” revealed that in almost all cases, research relied on the theoretical perspective of brand experience derived from the work of Schmitt (1999) and Brakus et al. (2009). To date, there is still only one definition, a single operationalization of the brand experience construct, and a single theoretical perspective through which it is approached (Andreini et al., 2018).

Contrary to the established conceptualization and operationalization of sensory brand experience, many other measurement scales related to the sensory aspect of brands seem to be too concrete; often, they are limited to a specific product category or a specific brand stimulus, which limits their generalizability and applicability. For example, Wiedmann et al. (2018) designed a scale for measuring the sensory impression of luxury hotels. Ebrahim et al. (2016) addressed the appearance perceptions of a brand. Khan et al. (2016) developed a measure involving corporate visual identity. Khan and Rahman (2016)’s scale includes an item pertaining to retail brand names. The scales proposed by Low and Lamb (2000) and Faircloth et al. (2001), and Chang and Chieng (2006) include only one sense-related item. Cho et al.’s (2015) sense-related items focus on visual elements of fashion brands. Thus, to date, no adequate holistic measurement model for multisensory marketing exists, and there has been a strong call for a shift toward a more holistic perspective (Roper et al., 2019).

Due to the overly abstract or restricted nature of extant scales, little is currently known about how to design sensory brand experiences that lead to high and sustainable brand strength and which provide customers with a high perceived value (Wiedmann et al., 2018). To better understand the mechanism by which to enhance brand experience via sensory brand stimuli, a new sensory brand experience scale with an appropriate level of abstraction needs to be developed. This scale should not be so abstract that it fails to provide useful diagnostic information, nor so specific that it cannot be generalized and used to compare different categories of brands. Therefore, the purpose of this research is to develop and validate such a sensory brand experience scale, specifically in the Chinese context. The following research questions are considered: What are the possible dimensions of sensory brand experience and; What are the specific items for each dimension of the sensory brand experience scale? A mixed-methods approach including both qualitative and quantitative methodologies is used to answer these questions.

## Study 1 Dimension Exploration and Item Generation

The objective of study 1 was to explore, through a literature review and consumer interviews, the possible dimensions of sensory brand experiences and to thereby propose an initial scale. First, we examined research related to sensory marketing, sensory branding, brand association, and brand experience, to identify factors that may influence consumers’ perceptions and assessments of a brand’s sensory quality. We then designed an exploratory qualitative study to assess consumers’ understanding of sensory brand experiences. Finally, we proposed an initial scale and possible items.

### Literature Review

The first dimension we identified from the literature is the volume of sensory brand stimuli. The quality of an experience increases with the number of senses that are addressed in a congruent way (Soars, 2009). Both the volume and the intensity of the applied sensory stimuli are decisive for the effective realization of brand experiences (Krishna, 2012). In addition, the greater the number of sensory memories activated, the stronger the connection established between brands and consumers (Lindstrom, 2005). This indicates that there are two factors that can influence consumers’ perceptions of the volume of sensory brand stimuli: the number of sensory channels and the number of sensory touchpoints.

The second dimension we propose is the uniqueness of sensory brand stimuli. Dewey (1963) suggested that experience should have the uniqueness that makes it stand out from the ordinary. Likewise, Pine and Gilmore (1988) described successful experiences as those that customers find unique, memorable, and enduring. According to Jackson and Fulberg (2003), the most important aspect to consider in creating a sound is the connection between that sound and the brand identity, where the sound should be distinctive, memorable, and spiritual.

The third dimension we posit is the consistency between sensory brand stimuli and consumer. Brand stimuli should be consistent with consumer’s consumption purpose. Consumers may have various objectives when shopping or consuming (Arnold and Reynolds, 2003; Rohm and Swaminathan, 2004). Brand stimuli should conform to the trends and fashions of the cultures and areas to which they are relevant. For example, people’s perception of smell is related to cultural differences (Spangenberg et al., 2006). Brand stimuli should also conform to consumer’s gender, race, and social class. For example, smells can be perceived as signs of femininity or masculinity (Spangenberg et al., 2006).

### Consumer’s Conceptions of Sensory Brand Experience

We conducted in-depth interviews with 20 consumers using the semi-structured interview method. Participants were first instructed to choose a brand that had made a strong sensory impression on them. They then wrote the brand down on a piece of paper, along with a description of their sensory experience. Next, participants were asked to choose a brand, in the same or a similar category, which had left a weak sensory impression on them. They then described the volume and uniqueness of the sensory brand stimuli for the brand, and the consistency between them and the sensory brand stimuli. Guided by our semi-structured questions, participants were asked not only to express their own concept of sensory brand experience, but also to think about it in the terms used by the interviewers. This allowed us to assess whether the consumers’ understanding was in line with our own, and how they perceived the difference between strong and weak sensory brand experience.

The interview data were analyzed and sorted by means of data coding and classification, with the purpose of extracting themes from this large amount of qualitative data (Lee, 1999). Two kinds of coding processes are widely used in qualitative research: one is completely open coding, and the other is to set coding variables according to existing theories. The second approach is considered more effective and realistic (Miles and Huberman, 1994), and was thus used in the current research to guide our coding. Specifically, we first defined related coding variables according to an exploration of the dimensions of sensory brand experience on the basis of the literature review. We then conducted a detailed analysis of the interview data using the codes derived. The qualitative analysis software ATLAS.ti 8 was used to facilitate coding. The findings of consumer interviews are consistent with the previous literature review. Example statements from the interviews in relation to the categories derived are presented in Table 1.

**TABLE 1:** Summary of dimensions of sensory brand experience.

**Core category**	**Definition**	**Subcategory**	**Example quotations from the interviews**
Volume	Volume of sensory brand stimuli		“In the supermarket, Master Kong accounted for a relatively large proportion of the shelves.”
Uniqueness	Uniqueness of sensory brand stimuli		“Apple is really unique in its appearance, and it can be distinguished clearly from other brands.”
Consistency	Consistency between sensory brand stimuli and consumer	Consistency with consumer’s consumption purpose	“I eat snacks mainly for relaxation. I want to be in a good mood when I relax, so if snacks are packaged prettily, I will feel happy.”
		Consistency with consumer’s culture	“Before the Chinese New Year, Coca Cola will launch an advertisement in line with the festival in red color, and I like it very much.”
		Consistency with consumer’s identity	“I like the glass bottle package of White Rabbit creamy candy. It seems not childish and a grownup like me can eat it comfortably in public.”
		Consistency with consumer’s personality	“The logo of Nokia strikes me as succinct. The font and the white and blue color give people a more rational feeling. I like this rational style because of my character. I am a rational person.”
		Consistency with consumer’s value	“I think inside its picture, its music, and its story expression, it has a feeling of being environmentally friendly and natural. I think highly of it because it is consistent with my values.”

### Item Generation

At this stage, we proposed an initial scale with specific dimensions and items. The goal was to generate and select items that truly describe brand sensory experience with good content validity. In order to generate these items, we combined related items from the literature and the concepts from the consumer interviews. The initial scale is presented in Table 2. Some items were revised from existing scales while others were designed by the authors. Three initial dimensions and 12 initial items were generated.

**TABLE 2:** Initial items of sensory brand experience.

**Dimension**	**Item**	**Source**
Volume	This brand mobilizes many of my senses	Self-compiled
	This brand provides me with a lot of sensory stimulation	Self-compiled
	This brand has few sensory elements	Self-compiled
Uniqueness	This brand is different from others in its sensory aspects	Adapted from Netemeyer et al. (2004) and Bruhn et al. (2012)
	This brand can stand out from other brands based on its sensory aspects	Adapted from Netemeyer et al. (2004) and Bruhn et al. (2012)
	I think this brand is unique in its sensory aspects	Adapted from Netemeyer et al. (2004) and Bruhn et al. (2012)
	Based on my sensory experience with this brand, it is highly distinguishable from other brands	Adapted from Netemeyer et al. (2004) and Bruhn et al. (2012)
Consistency	The sensory characteristics of this brand are consistent with my consumption objective	Adapted from Ellen et al. (2000)
	The sensory characteristics of this brand are suitable for this locality	Adapted from Ellen et al. (2000)
	The sensory characteristics of this brand are consistent with my identity (age, gender, race, social class, etc.)	Adapted from Ellen et al. (2000)
	The sensory characteristics of this brand are consistent with my personality	Self-compiled
	The sensory characteristics of this brand are consistent with my values	Self-compiled

## Study 2 Preliminary Examination of Items and Exploration of Scale Validity

### Pilot Study

The objective of the pilot study was to identify, for use in the subsequent main study, certain brands that generate a strong sensory experience and others that generate a weak sensory experience. To do so, we used the method of consumer nomination, for which 43 consumers were recruited as participants. Each participant was asked to think of three product categories, and for each category, select one brand that generates strong sensory experience and one brand that generates weak sensory experience. The brands that participants identified for the strong sensory experience category included global brands such as Apple, Benz, Coca-Cola, Haier, Huawei, Lenovo, MUJI, Panasonic, and Volkswagen, as well as some local brands. The brands that participants considered for the weak sensory experience category included global brands such as Apple, Haier, Lenovo, ONLY, Pepsi Cola, Rejoice, and Samsung, as well as some local brands. We noticed that some brands (Apple, Haier, Lenovo) appeared under both the strong and weak designations, reflecting that these brands made discrepant impressions on different consumers, so we did not include them in the next main study. We retained the other 15 brands with the highest frequency of mentions (10 experiential brands and 5 non-experiential brands).

### Main Study

The objective of the main study was to preliminarily examine the items and to test the validity of the scale. To this end, the scale shown in Table 2 was applied as the measurement tool. A new consumer sample was recruited and divided into four groups. We designed four different versions of the questionnaire for each group. Each version contained five well-known brands, including both strong and weak brands in terms of sensory brand experience according to consumers’ perceptions identified in the pilot study. A strong brand (Huawei) was included in each version of the questionnaire in order to test the consistency of grading among the four groups. Each participant received one of the four editions, using a seven-point Likert scale (“1” = “strongly disagree,” “7” = “strongly agree”) to evaluate the items. In total, 279 questionnaires were distributed, of which 220 were returned (first group: 65 distributed and 54 returned; second group, 71 distributed and 55 returned; third group, 67 distributed and 55 returned; fourth group, 76 distributed and 56 returned). We used IBM SPSS Statistics 25 to analyze the data.

Based on the survey data, the internal consistency and stability of the scale were evaluated to confirm the scale’s reliability. According to the statistical results of item-total correlation, the item “This brand has few sensory elements” made a negative contribution to the Cronbach’s α value. This item was a reverse question; the wording was originally intended to reduce common method variance, but it may have led to new problems. Rather than simply deleting it, in the next study we revised the item into a positive sentence to remeasure this aspect.

Huawei was identified in our pilot study as a typical strong brand with respect to sensory experience. In the main study, Huawei received a high score in all four groups, which illustrates that respondents used the scale in a similar way.

To check the criterion validity of the scale preliminarily, we calculated the mean value of the items for each of the brands. The descriptive statistics indicated that the mean scores of all brands vary greatly. The average scores of the brands identified in the pilot study as having strong sensory experience were generally higher than those with weak sensory experience. The means of experiential brands ranged from 5.1656 to 5.5779, in contrast, the means of non-experiential brands were lower and ranged from 4.3805 to 5.1241. In order to conduct a more accurate analysis of the score differences among brands, we conducted a paired-samples *t*-test of 105 combinations and ran pairwise comparisons among all brands involved in the survey. The results show that most of the brands differed from each other, since the mean score for each brand was significantly different (*p* < 0.05). Thus, it was verified that the scale could indeed be used as a tool to distinguish the sensory quality of brands.

## Study 3 Formal Scale – Determination and Validation

The goal of this study was to further examine the items, verify the dimensions, test the reliability and validity of the scale, and finally determine the formal scale. IBM SPSS Statistics 25 and IBM SPSS Amos 25 were used to analyze the data in study 3.

### Study and Item Design

We changed the item “This brand has few sensory elements” into a positive sentence – “This brand has many sensory elements” – and kept other items in Table 2 unchanged. To test the stability of the scale, we applied new brands and used a new respondent sample. This helped us to test whether the response to the scale items was independent of particular brands or participants, so that the tool could be used for general assessment of brand sensory experience. Therefore, in study 3 we selected 15 well-known brands, including both global and local ones, mainly based on expert judgment; we then divided these randomly into three groups of five brands each. The 15 brands were:

• Group 1: Adidas, Burberry, Disney, Lego, Johnson & Johnson
• Group 2: ANTA, HYX, L’Oréal, Nestle, Starbucks
• Group 3: Apple, Dell, HSTYLE, IKEA, LETV

For each group we assigned more than 50 surveys; in total, 227 questionnaires were distributed and 164 returned. Since each participant provided responses with reference to five brands, the sample size equaled 820. A seven-point Likert scale (“1” = “strongly disagree,” “7” = “strongly agree”) was used to evaluate the items. A preliminary exploratory factor analysis showed that some items had large factor loadings in more than one factor, which indicated that these items were vague in meaning. Deleting these items did not harm the reliability of the scale overall; thus, following their deletion, 10 items remained.

### Dimension Exploration

We conducted exploratory factor analysis again against the 10 items in order to explore the possible dimension structure of the new scale. The KMO measure and Bartlett’s test of sphericity were used to test whether the data were suitable to perform exploratory factor analysis (EFA). The KMO index was 0.933, and Bartlett’s test of sphericity was significant at a level of 0.000, which meant that EFA was suitable for the data. Three factors were extracted that cumulatively explained 81.570% of the total variance. After setting not displaying low factor loadings (<0.5), the rotated component matrix indicated a highly ordered distribution. The first factor included three items related to the volume of sensory brand stimuli, so we named it “volume.” The second factor included three items related to the uniqueness of sensory brand stimuli, so it was named “uniqueness.” The third factor included four items related to the consistency between sensory brand stimuli and consumer and was thus named “consistency.” In this way, we obtained a new scale comprising three dimensions and 10 items (Table 3). The Cronbach’s α index for the scale was 0.943, suggesting good internal consistency.

**TABLE 3:** Formal scale of sensory brand experience.

**Dimension**	**Item**	**Factor loading**
Volume	V1: This brand mobilizes many of my senses	0.830
	V2: This brand provides me with a lot of sensory stimulation	0.815
	V3: This brand has many sensory elements	0.720
Uniqueness	U1: This brand is different from others in its sensory aspects	0.759
	U2: This brand can stand out from other brands based on its sensory aspects	0.799
	U3: I think this brand is unique in its sensory aspects	0.794
Consistency	C1: The sensory characteristics of this brand are suitable for this locality	0.748
	C2: The sensory characteristics of this brand are consistent with my identity (age, gender, race, social class, etc.)	0.797
	C3: The sensory characteristics of this brand are consistent with my personality	0.798
	C4: The sensory characteristics of this brand are consistent with my values	0.801

### Scale Validation

On the basis of the above exploratory work, we performed a first-order three-factor confirmatory factor analysis (CFA) and a second-order CFA to finally determine the dimensions of the scale and to validate its reliability and validity. Before constructing the measurement model, the normality of the data and common method variance were assessed.

#### Normality Assessment

The parameters were estimated using the maximum likelihood method. Studies have proven that the parameter estimation results of the maximum likelihood method are more accurate compared to those of other methods in most situations. However, the premise of parameter estimation using the maximum likelihood method is that data must conform to the assumption of multivariate normality; thus, it is necessary to carry out a normality test for observed data before conducting structural equation modeling (SEM). Table 4 shows the result of the normality assessment. In SEM, if the absolute value of skewness of the observed variable of sample data is greater than 3, or the kurtosis coefficient is greater than 8, the observed data may deviate from the normal distribution, especially when the kurtosis coefficient is greater than 20, indicating that the kurtosis of the data is significantly different from normal (Kline, 1998). In this study, the absolute values of skewness of all variables were less than 3 and the kurtosis values of all variables were less than 8; thus, data did not deviate from normal distribution and can be said to meet the requirements of multivariate normality.

**TABLE 4:** Assessment of normality.

**Variable**	**Min.**	**Max.**	**Skewness**	**Kurtosis**
V1	1.000	7.000	–0.647	0.393
V2	1.000	7.000	–0.611	0.329
V3	1.000	7.000	–0.685	0.484
U1	1.000	7.000	–0.582	0.318
U2	1.000	7.000	–0.808	0.678
U3	1.000	7.000	–0.645	0.414
C1	1.000	7.000	–0.678	0.494
C2	1.000	7.000	–0.761	0.499
C3	1.000	7.000	–0.708	0.522
C4	1.000	7.000	–0.738	0.645
Multivariate				56.736

#### Evaluation of Common Method Variance

Method variance is the variance of a systematic error caused by the measurement method. Common method variance (CMV) refers to the fact that the overlap of variance between two variables is due to the use of similar measurement tools, rather than representing the true relationship between the underlying constructs (Teo, 2011). Using the same method to measure each variable is likely to produce spurious correlations between variables; this is common in surveys. CMV can be controlled via two methods: process control and statistical control. Process control is to control the research design in advance, according to various possible sources of method variance. The current study used the design of anonymous filling to do the process control. Statistical control entails reducing the influence of method variance on the results of in-test or inter-test research through statistical means.

**FIGURE 1:**
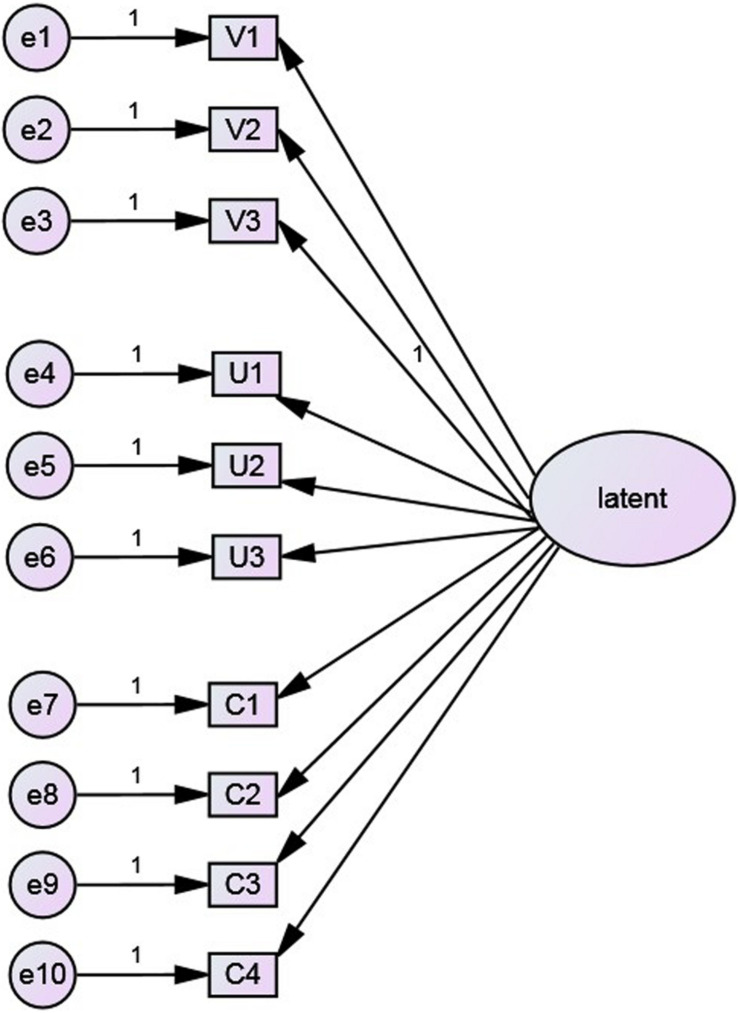
The first-order single factor model for testing CMV.

Two statistical control methods were used in this study. First, Harman’s single factor test (Hu and Bentler, 1999) was used to investigate CMV. A first-order single-factor model was constructed (Figure 1), in which all items were loaded on a single latent factor. Based on the overall model fit test of CFA, the goodness of fit indices of the model were obtained (Table 5). According to the standard or critical value of adaptation, it can be judged that the single-factor model and data do not fit, indicating that common method variance is not an obvious issue.

**TABLE 5:** Fit indices of the first-order single-factor model.

	**CMIN**	**DF**	**CMIN/DF**	**GFI**	**AGFI**	**RMR**	**RMSEA**	**NFI**	**RFI**	**IFI**	**TLI**	**CFI**	**PGFI**
First-order single-factor model	995.888 *p* = 0.000	35	28.454	0.760	0.623	0.105	0.183	0.851	0.809	0.856	0.814	0.855	0.484
Recommended standard	*p* > 0.05		<3.0	>0.90	>0.90	<0.05	<0.08	>0.90	>0.90	>0.90	>0.90	>0.90	>0.50

Second, the CMV of the scale was tested using single method-factor approaches, which are mainly used for CMV control when applying the same method to measure the studied constructs. The analysis was performed using SEM. In structural equations, all items of the measured constructs load not only on the factors to which they belong, but also on a single common latent method factor. It is assumed that the latent method factor cannot be measured, and the loadings of the method factor only come from items of the measurement constructs. The variance analysis idea is that the score variance of the items of the measurement construct can be divided into corresponding trait variance, method variance, and measurement error.

**FIGURE 2:**
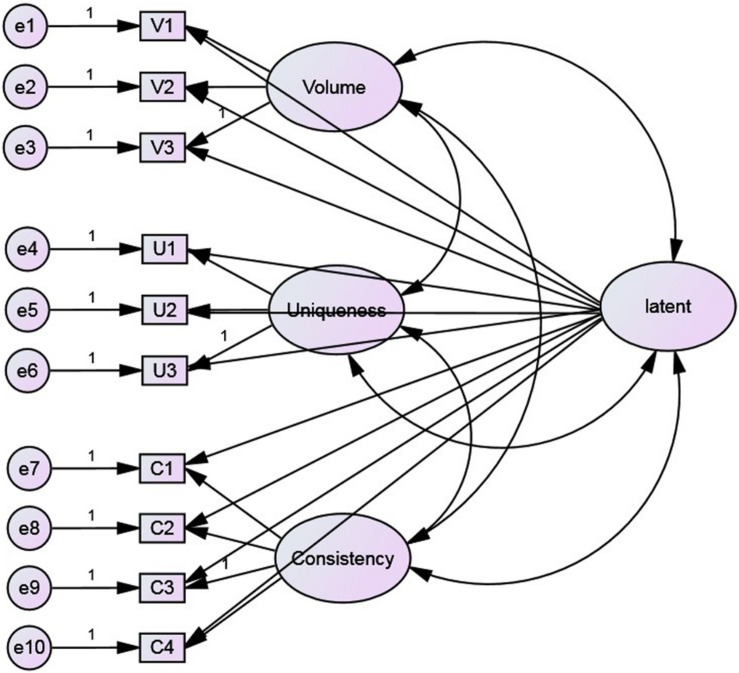
The single method-factor approaches model for testing CMV.

In this study, on the basis of the first-order three-factor model, a method factor affecting all items was added. This factor is not related to any latent trait factors, meaning that each item is affected by a trait factor, a method factor, and a residual. The single-method latent factor model constructed is shown in Figure 2. From the parameter estimation results, negative error variances can be identified, indicating that the model is incorrect. In addition, several fit indices do not reach acceptable standards, especially the parsimony goodness of fit index (Table 6). These results indicate that common method variance is not a significant issue in this study and does not affect the structural validity of the scale.

**TABLE 6:** Fit indices of the single method-factor approach model.

	**CMIN**	**DF**	**PGFI**	**PNFI**	**PCFI**
Single method-factor approaches model	37.003 *p* = 0.024	22	0.397	0.486	0.488
Recommended standard	*p* > 0.05		>0.50	>0.50	>0.50

#### Goodness of Fit of the First-Order Three-Factor Model

Goodness of fit is used to evaluate the degree of consistency between the assumed path analysis model and the collected data. Many scholars (Bagozzi and Yi, 1988) believe that the following three aspects should be considered in evaluating whether the hypothesized model fits the actual data: (1) preliminary fit criteria, wherein, before estimating the fit degree of the overall model, the researcher should test whether the model violates the estimate and check the feasibility of the parameter estimate; (2) overall model fit, which tests the external quality of the model; and (3) fit of the internal structural model, which tests the intrinsic quality of the model.

**FIGURE 3:**
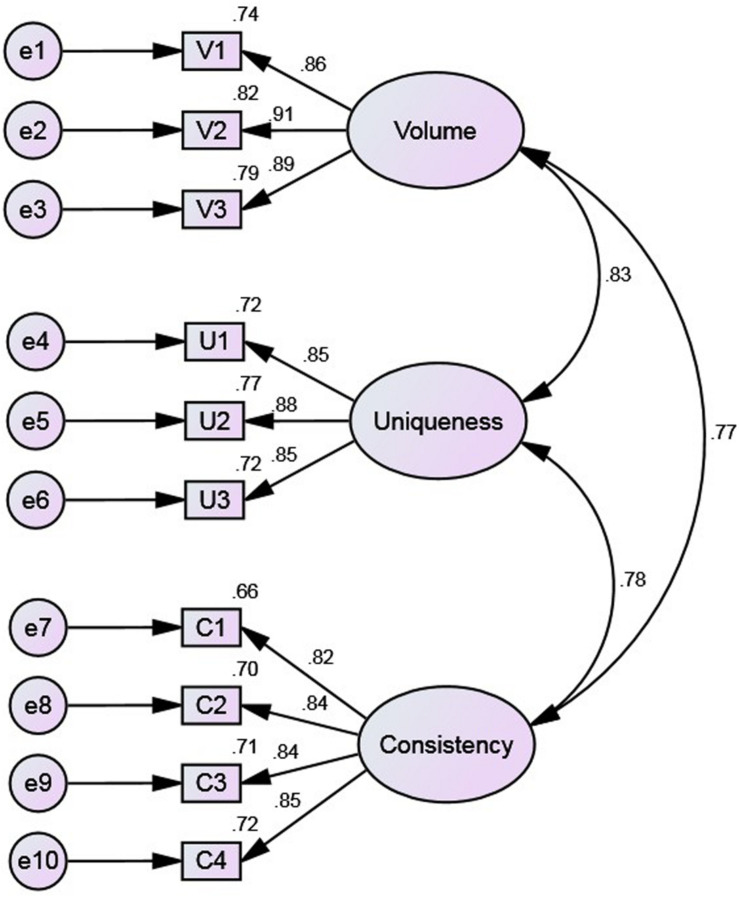
The first-order model.

We first constructed a first-order three-factor model (Figure 3), then evaluated its goodness of fit according to the criteria above. The three factors considered are: volume, uniqueness, and consistency.

In terms of preliminary fit criteria, no negative error variances are noted in the model estimation parameters, which are all significant. There are no large standard errors, and their values are 0.023–0.081. The factor loadings of the items are 0.815–0.905. This indicates that the preliminary fit criteria of the model are good and there are no model specification mistakes.

In terms of overall model fit (Table 7), all the indicators except the chi-square value meet acceptable standards. Considering the large sample size (820), the chi-square value to the degrees of freedom ratio was 3.631; since this is close to 3, this index is also acceptable. Thus, on the whole, the first-order three-factor model proposed in this study fit the actual observed data well; that is, the external quality of the model is good.

**TABLE 7:** Fit indices of the first-order three-factor model.

	**CMIN**	**DF**	**CMIN/DF**	**GFI**	**AGFI**	**RMR**	**RMSEA**	**NFI**	**RFI**	**IFI**	**TLI**	**CFI**	**PGFI**	**PNFI**	**PCFI**
Model	116.196 *p* = 0.000	32	3.631	0.973	0.954	0.031	0.057	0.983	0.976	0.987	0.983	0.987	0.566	0.699	0.702
Recommended standard	*p* > 0.05		<3.0	>0.90	>0.90	<0.05	<0.08	>0.90	>0.90	>0.90	>0.90	>0.90	>0.50	>0.50	>0.50

The fit of the internal structural model represents the reliability and validity of the measurement model. The squared multiple correlation coefficient (R^2^) values of all items are higher than 0.50, ranging between 0.665 and 0.819; the composite reliability (CR) values of the latent variables are greater than 0.60, ranging between 0.8939 and 0.9146; and the Cronbach’s α values of all factors are greater than 0.8, ranging between 0.893 and 0.914 (Table 8). These indicators show that the reliability of the measurement model is very good. The standardized loadings are all greater than 0.8 and significant (Table 8), while the average variance extracted (AVE) values of the latent variables are all greater than 0.50, ranging between 0.7003and 0.7812 (Table 9). This indicates that the latent variables of the measurement model have good convergent validity. In addition, the square roots of the AVE of all latent variables are greater than the correlation coefficient between latent variables (Table 9), indicating that the scale has good discriminant validity. Finally, the estimated parameters are all significant; the absolute values of the standardized residuals are all less than 2.58, and the maximum absolute value is 1.463. In conclusion, the intrinsic quality of the model is very good.

**TABLE 8:** Reliability of the first-order three-factor model.

**Factor**	**Item**	**Standardized loading**	***t*-value**	***R*^2^**
Volume	V1	0.858	33.709	0.736
Cronbach’s α = 0.914	V2	0.905	37.308	0.819
CR = 0.9146	V3	0.888	–	0.788
Uniqueness	U1	0.850	30.148	0.722
Cronbach’s α = 0.893	U2	0.875	31.532	0.766
CR = 0.8939	U3	0.851	–	0.725
Consistency	C1	0.815	28.027	0.665
Cronbach’s α = 0.903	C2	0.837	29.170	0.700
CR = 0.9033	C3	0.845	–	0.714
	C4	0.850	29.869	0.722

**TABLE 9:** Validity of the first-order three-factor model.

	**Volume**	**Uniqueness**	**Consistency**
Volume	0.884		
Uniqueness	0.833	0.859	
Consistency	0.770	0.780	0.837
AVE	0.7812	0.7374	0.7003

#### Goodness of Fit of the Second-Order Model

**FIGURE 4:**
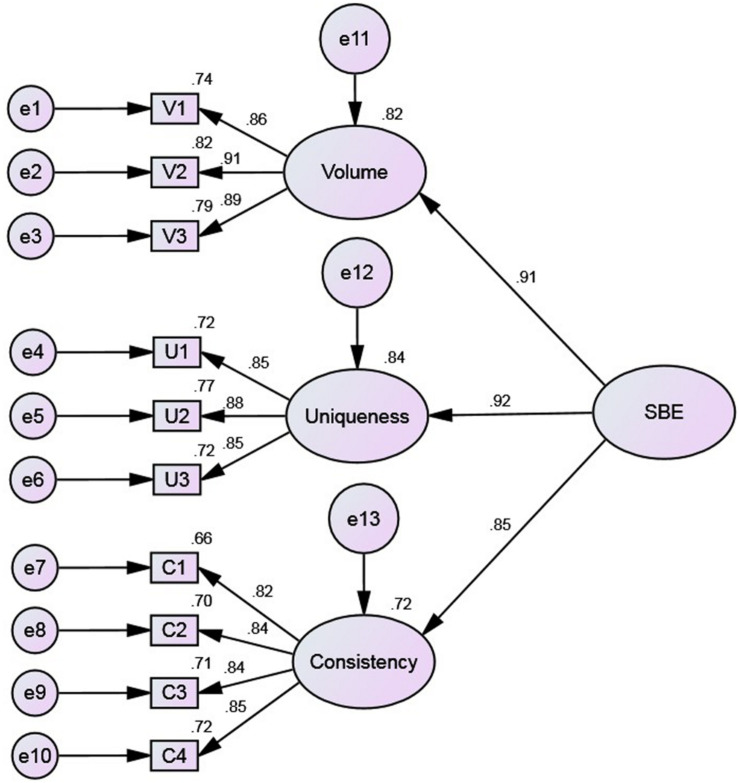
The second-order model.

The second-order factor model (Figure 4) is based on the first-order three-factor model, but the three factors constitute a higher-order factor. This model was constructed on the basis of the high correlation among the three factors found in the first-order three-factor model analysis (Table 9), which indicated that it was possible to have a common higher-order factor behind the three factors. The second-order factor was named sensory brand experience (SBE).

In terms of preliminary fit criteria, no negative error variances are noted in the model estimation parameters, which are all significant. There are again no large standard errors, and their values are 0.023–0.079. The factor loadings of the items are 0.815–0.919. This indicates that the preliminary fit criteria of the model are good and there are no model specification mistakes.

In terms of overall model fit, the goodness of fit is the same as in the first-order three-factor model (Table 7) since there are only three first-order factors. The second-order model again fits the actual observed data well; that is, the external quality of the model is good.

In terms of the fit of the internal structural model, the squared multiple correlation coefficient (R^2^) values of all items are higher than 0.50, ranging between 0.665 and 0.844; the CR values of the latent variables are greater than 0.60, ranging between 0.8939 and 0.9215; and the Cronbach’s α values of all factors are greater than 0.8, ranging between 0.893 and 0.914 (Table 10). These indicators demonstrate that the reliability of the measurement model is very good. In addition, the standardized loadings are all greater than 0.8 and significant, while the AVE values of the latent variables are all greater than 0.50, ranging between 0.7003 and 0.7966 (Table 10). This indicates that the latent variables of the measurement model have good convergent validity. The previous analysis of the first-order three-factor model indicates that the scale has good discriminant validity and the estimated parameters are all significant, with absolute values of standardized residuals less than 2.58, and a maximum absolute value of 1.463. In conclusion, the intrinsic quality of the model is very good.

**TABLE 10:** Reliability and validity of the second-order model.

**Factor**	**Item/factor**	**Standardized loading**	***t*- value**	***R*^2^**	**Cronbach’s α**	**CR**	**AVE**
Volume	V1	0.858	33.709	0.736	0.914	0.9146	0.7812
	V2	0.905	37.308	0.819			
	V3	0.888	–	0.788			
Uniqueness	U1	0.850	30.148	0.722	0.893	0.8939	0.7374
	U2	0.875	31.532	0.766			
	U3	0.851	–	0.725			
Consistency	C1	0.815	28.027	0.665	0.903	0.9033	0.7003
	C2	0.837	29.170	0.700			
	C3	0.845	–	0.714			
	C4	0.850	29.869	0.722			
SBE	Volume	0.907		0.823		0.9215	0.7966
	Uniqueness	0.919	23.222	0.844			
	Consistency	0.850	22.010	0.722			

## Discussion

The aim of this study was to create a useful instrument by which to measure sensory brand experience. This entailed a process of scale development and validation. In study 1, we carried out a qualitative study to explore possible dimensions and items for the scale by conducting semi-structured interviews. Several dimensions and items were proposed by combining a literature review and the consumer interviews. In study 2, we preliminarily examined the items and tested the validity of the scale. The results show that, according to our scale, most of the brands used in the study differ from one another in terms of sensory brand experience; this indicates that the scale is suitable for differentiating strong from weak sensory brand experience. The scale thus has the potential to be used to evaluate the sensory quality of brands. In study 3, we further examined the items, verified the dimensions, tested the reliability and validity of the scale, and finally determined a formal scale.

The sensory brand experience scale in this study contains 10 items. These items were classified into three dimensions according to the results of EFA and CFA analysis (Tables 2, 3), which coincided with the three core categories of the content analysis results of the qualitative study (Table 1). The three dimensions represent, respectively, three important factors that may influence consumers’ perceptions and evaluations of the sensory quality of brands: the volume of sensory brand stimuli, the uniqueness of sensory brand stimuli, and the consistency between sensory brand stimuli and consumer. The reliability and validity of the scale are high, while the first-order three-factor model and the second-order model both fit the data well. Therefore, the models can be considered valid and can be used in subsequent studies.

The scale also conforms to previous concepts and theory of sensory brand experiences. To check this, we compared our scale to the sensory dimension of brand experience scale proposed by Brakus et al. (2009). Brakus et al. (2009) mentioned that their brand experience scale focuses on the level of experience stimulated by the brand in various dimensions. Their scale is thus highly abstract, measuring only whether and to what extent consumers have experience on a brand. Sensory experience is only one dimension of their scale and comprises only three items. The sensory brand experience scale developed in the present study is more specific and has a medium degree of abstraction.

We combined the items of our scale with Brakus et al.’s (2009) three items of sensory brand experience to conduct an EFA. Results of KMO and Bartlett’s test indicated that factor analysis was appropriate. Two factors were extracted with their eigenvalues greater than 1, which explained 73.479% of the total variance. After rotation, all items regularly loaded on two factors. Factor 1 contained two dimensions of our scale, volume and uniqueness, as well as one item from Brakus et al. (2009), “This brand makes a strong impression on my visual sense or other senses.” Factor 2 contained one dimension of our scale, consistency, and two items from Brakus et al. (2009), “I find this brand interesting in a sensory way,” and “This brand does not appeal to my senses.” Factor 1 reflected the strength of sensory brand experience, while factor 2 reflected the favorability of sensory brand experience. This illustrates that our scale has the same theoretical meaning as Brakus et al.’s (2009) conception with respect to sensory brand experience. However, with 10 items and three dimensions our scale is more specific and can reveal a greater amount of information about sensory brand experience, so can serve as a useful diagnostic tool for guiding brands in promoting their sensory quality.

In conclusion, the sensory brand experience scale proposed in this study conforms to the scale suggested by Brakus et al. (2009) but is more concrete. At the same time, from a holistic perspective, it still has sufficient abstraction and is not limited to a specific product category or a specific brand stimulus, which ensures its generalizability and applicability.

### Implications

The current findings have important implications for both practical and research settings. In an environment where consumers are becoming increasingly demanding, homogeneous products are continuously emerging, and advertising campaigns are becoming increasingly fierce, sensory brand experience has become a powerful tool to make brands stand out. However, in sensory branding practice, many brands encounter difficulties due to a lack of effective theoretical guidance. The ways in which consumers evaluate sensory brand experience, and what dimensions should be included in its structure, have been largely ignored by research to date. One reason for this may be that, compared with other brand research topics, sensory brand measurement scales are very scarce. Therefore, there is an urgent need to develop sensory brand evaluation tools.

This study evaluated sensory brand experience from the perspective of consumers. It discussed and defined the measurement dimensions of sensory brand experience. The sensory brand experience evaluation scale developed was shown to have good reliability and validity and represents a practical measurement and evaluation tool for sensory brand building. The scale can be used as a measurement and diagnostic tool for brands, serving as a focal point for sensory branding practice. The scale was designed for use as a general measurement tool and is therefore suitable for all kinds of brands. It is also particularly useful for tracking brands’ sensory experience trends. According to our findings, brand comparisons can also be conducted to rank the sensory performance of brands within an industry or between different industries, which will help to improve brands’ competitiveness. As an example of the application of the scale, the brands used in study 3 were ranked in terms of sensory brand experience (Table 11).

**TABLE 11:** Sensory brand experience (SBE) ranking (for brands in study 3).

**Brand**	**SBE**	**Volume**	**Uniqueness**	**Consistency**
Adidas	5.56	5.57	5.55	5.55
Apple	5.55	5.81	5.57	5.34
Disney	5.42	5.60	5.59	5.15
Nestle	5.21	5.30	5.18	5.15
LETV	5.12	5.27	4.97	5.13
Burberry	5.07	4.90	5.20	5.10
Johnson & Johnson	5.05	5.00	5.07	5.06
Lego	5.03	4.86	5.14	5.06
Starbucks	4.99	5.07	5.19	4.77
IKEA	4.98	4.97	4.93	5.02
Dell	4.96	4.91	4.84	5.07
L’Oréal	4.86	4.74	5.00	4.83
ANTA	4.64	4.45	4.63	4.81
HYX	4.61	4.24	4.71	4.82
HSTYLE	4.43	4.39	4.35	4.53

## Data Availability Statement

The datasets generated for this study are available on request to the corresponding author.

## Ethics Statement

The studies involving human participants were reviewed and approved by the Shanghai Urban Construction Vocational College. The patients/participants provided their written informed consent to participate in this study.

## Author Contributions

FG generated ideas for the study, conducted the literature review, and drafted the manuscript. XL participated in the discussions and helped to draft the manuscript. All authors contributed to the article and approved the submitted version.

## Conflict of Interest

The authors declare that the research was conducted in the absence of any commercial or financial relationships that could be construed as a potential conflict of interest.
